# Native Valve Infective Endocarditis Caused by *Histoplasma capsulatum* in an Immunocompetent Host: The First Case in Asia and Literature Review in Asia and Australia

**DOI:** 10.1155/2021/9981286

**Published:** 2021-06-21

**Authors:** Kamalas Amnuay, Chayatat Sirinawin, Nonthikorn Theerasuwipakorn, Pairoj Chattranukulchai, Chusana Suankratay

**Affiliations:** ^1^Division of Infectious Diseases, Department of Medicine, Faculty of Medicine, Chulalongkorn University, Bangkok, Thailand; ^2^Division of Cardiothoracic Surgery, Department of Surgery, Faculty of Medicine, Chulalongkorn University, Bangkok, Thailand; ^3^Division of Cardiovascular Medicine, Department of Medicine, Faculty of Medicine, Chulalongkorn University, Bangkok, Thailand

## Abstract

**Background:**

Infective endocarditis caused by the dimorphic fungus *Histoplasma capsulatum* is extremely rare, occurring predominantly in individuals with prosthetic heart valves and HIV infection. To our knowledge, no case of *H. capsulatum* native valve endocarditis has been reported in Asia. *Methodology*. A descriptive study was carried out at King Chulalongkorn Memorial Hospital, Bangkok, Thailand, in 2020.

**Results:**

A previously healthy 34-year-old man developed fever, umbilicated skin lesions, oral ulcers, hoarseness of voice, severe weight loss, and progressive dyspnea over the course of one week. Facial umbilicated papules, nodular ulcers in his tongue and palate, a diastolic rumbling murmur at the mitral valve, diffuse fine crackles in both lungs, and engorged neck veins were detected during the examination. Skin scraping of the facial lesion revealed both extracellular and intracellular yeasts with buddings, 2–4 *μ*m in size on Wright's stain. Transthoracic echocardiography demonstrated a left ventricular ejection fraction of 54 percent, severe rheumatic mitral stenosis, and multiple oscillating masses in the anterior mitral valve leaflet ranging in dimension from 1.5 to 2.4 cm. The HIV antibody test was negative. *H. capsulatum* endocarditis was diagnosed, and liposomal amphotericin B was administered. Due to cardiogenic shock, emergency open-heart surgery was conducted one day after admission. However, he died of multiorgan failure four days after the operation. The skin and vegetation cultures finally grew *H. capsulatum* after 1 week of incubation.

**Conclusions:**

To date, there has been handful of cases of *H. capsulatum* native valve endocarditis in non-HIV-infected patients. We report herein the first case in Thailand. Umbilicated skin lesions, especially combined with oral mucosal lesions, are a clinical clue that leads to the correct diagnosis of the causative organism.

## 1. Introduction

One of the thermally dimorphic fungi, *Histoplasma capsulatum*, can cause opportunistic infections among immunocompromised hosts, especially in AIDS patients and organ transplant recipients. The disease is endemic in North and Central America and some parts of South America, Africa, Australia, and Asia. Thailand is an endemic area as well [1]. Histoplasmosis presents as a wide spectrum of forms, varying from mild localized to disseminated infection [2–5]. Infective endocarditis caused by *H. capsulatum* is extremely rare, occurring predominantly in patients with prosthetic heart valves and HIV infection. To our knowledge, there have been no reported cases of native valve endocarditis caused by *H. capsulatum* in Asia. We provide the first case of *H. capsulatum* endocarditis in a non-HIV-positive patient in Thailand and a review of the English literature for *H. capsulatum* endocarditis in Asia.

## 2. Case Report

**Figure 1:**
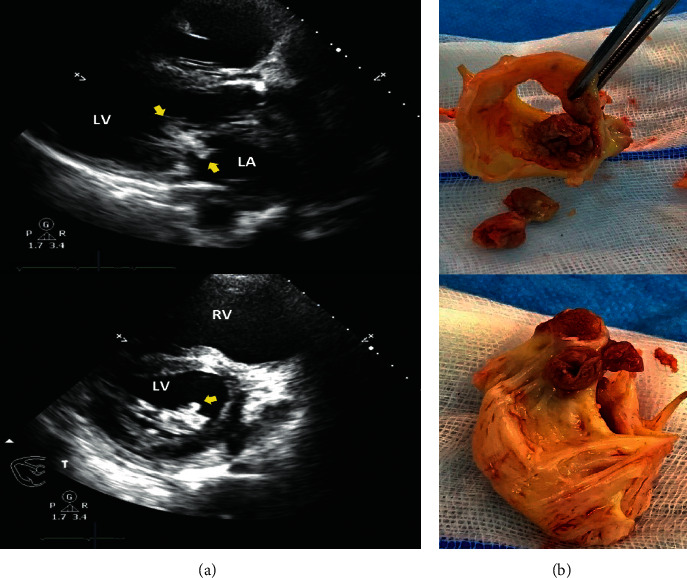
(a) Transthoracic echocardiogram showing multiple oscillating masses (yellow arrows) at anterior mitral valve leaflet with severe rheumatic mitral stenosis. (b) Gross findings of the resected mitral valve showing three large vegetations at anterior mitral valve leaflet. LV, left ventricle; LA, left atrium; RV, right ventricle.

**Figure 2:**
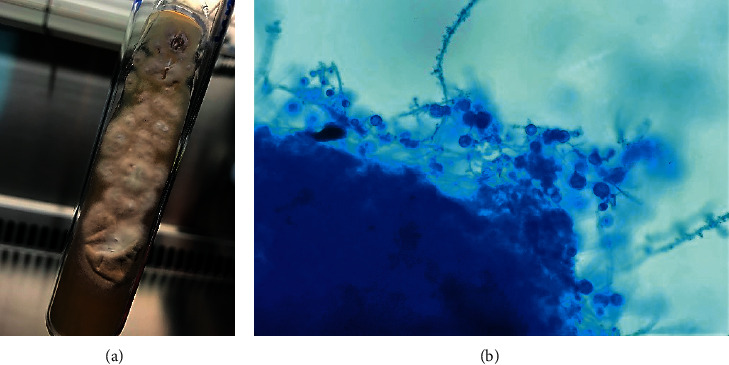
(a) The colonies of mold form. (b) Lactophenol cotton blue stain showing hyphae with numerous tuberculate macroconidia.

A 34 year-old Thai man from Prajin Buri, a province in rural East Thailand, presented with a 4-month history of low-grade fever and multiple discrete painful umbilicated erythematous papules on his face, as well as a 40 kg weight loss from his previous normal weight of 110 kg and height of 170 cm. Two weeks prior to admission (PTA), he developed odynophagia, painful nodular ulcers on his tongue, and hoarseness of his voice. One week PTA, he had dyspnea on exertion. His medical history was unremarkable, and he denied using any pills, herbs, or intravenous drugs. His occupation was that of a car mechanic. He mentioned that he had not traveled to other locations in the previous three months. Physical examination revealed blood pressure of 90/50 mmHg, regular heart rate of 110/min, respiratory rate of 24/min, and body temperature of 38.3°C, mildly pale conjunctivae, multiple papules with central necrosis on his face painful nodular ulcers on his tongue and palate, diffuse fine crackles in both lungs, jugular vein engorgement, a positive left ventricular heave and a grade III/VI diastolic rumbling murmur at mitral valve area, hepatosplenomegaly, and mild pedal edema. The complete blood count (CBC) analysis showed a Hb of 11.1 g/dL, white blood cell of 5,350 × 103/*μ*L, and platelets of 140,000/*μ*L. Blood chemistries showed urea nitrogen of 56 mg/dL, creatinine of 1.98 mg/dL, total bilirubin of 4.9 mg/dL, direct bilirubin of 3.09 mg/dL, aspartate aminotransferase of 42 U/L, alanine aminotransferase of 25 U/L, alkaline phosphatase of 147 U/L, albumin of 3.1 g/dL, corrected calcium of 13.4 mg/dL, phosphorus of 3.1 mg/dL, intact parathyroid hormone of <1.2 (normal: 15–65) pmol/L, and 1,25-OH2 vitamin D of 152 (normal: 19.9–79.3) pg/mL. Anti-HIV serology testing showed negative results. Cardiomegaly with pulmonary congestion was seen on chest radiography. Transthoracic echocardiography indicated a left ventricular ejection fraction of 54%, moderate aortic regurgitation, severe rheumatic mitral stenosis, and multiple oscillating masses on the anterior mitral valve leaflet ranging from 1.5 cm to 2.4 cm in diameter (Figure 1(a), yellow arrows). Skin scraping of the facial lesion exhibited both extracellular and intracellular yeast-like organisms with buddings, 2–4 *μ*m in size, on Wright's stain, most likely *Histoplasma*. As a result, disseminated histoplasmosis and endocarditis were diagnosed, and 3 mg/kg/day intravenous liposomal amphotericin B was administered immediately. The patient had a profound cardiogenic shock and atrial fibrillation one day after being admitted to the hospital, and echocardiography indicated a mechanical obstruction of the mitral valve orifice caused by the vegetation. Three large vegetations on the anterior mitral valve leaflet were identified during emergency open-heart surgery (Figure 1(b)), and a mechanical prosthetic mitral valve was placed. Histopathology examination of the vegetation showed many yeast-like organisms. However, the patient's condition deteriorated, and he died four days after the operation from intractable multiorgan failure. The sputum and vegetation cultures grew *H. capsulatum* (Figures 2(a) and 2(b)) after 1 week of incubation.

## 3. Discussion

To our knowledge, there have been no reported cases of native valve endocarditis caused by *H*. *capsulatum* in Asia. We report herein the first case of *H. capsulatum* endocarditis in an immunocompetent host in Asia. A retrospective study in 1965–1995 and the review study in 1995–2000 showed that *Candida* and *Aspergillus* were the most common causative agents of fungal endocarditis. Cardiac structural abnormalities, such as rheumatic valvulopathy and the prosthetic valve, are common predisposing factors for fungal endocarditis. Endocarditis caused by *H. capsulatum* was unusually infrequent [3, 6]. Despite the fact that our patient acquired *Histoplasma* endocarditis without obvious exposure, the concurrent rheumatic mitral stenosis may have been a significant predisposing factor.

The cutaneous manifestations of histoplasmosis may be due to either direct skin inoculation or hematogenous dissemination which is much more common [7]. They might appear as macules, papules, nodules, vesicles, ulcers, subcutaneous lesions, and molluscum-like lesions in immunocompetent hosts. [7] In AIDS patients, however, molluscum-like lesions are the most common type [8–10]. The differential list of the causative agents causing molluscum-like lesions includes *Talaromyces marneffei*, *Cryptococcus neoformans*, *Mycobacterium avium* complex [11], and *Mycobacterium abscessus* [12]. Facial molluscum-like lesions were detected in our patient, which are uncommon in non-HIV-positive people. In Thailand, mucocutaneous involvement is more common in histoplasmosis than in other endemic mycosis and cryptococcosis, particularly in immunocompetent hosts [7, 13, 14]. Our patient had oropharyngeal lesions as well.

The manifestations of infective endocarditis caused by fungus include dyspnea, fever, fatigue, and weight loss [15]. The skin signs including Janeway's lesions, Osler's nodes, and splinter hemorrhage are rarely present. Our patient had developed progressive dyspnea, fever, and was losing weight. He did not have skin signs of embolic and vascular phenomena of infective endocarditis.

We hypothesize that infective endocarditis in our patient was the consequence of the dissemination of *H. capsulatum* due to the mucocutaneous manifestations (dissemination) occurring many months prior to the symptoms of heart failure (endocarditis). In addition, most patients with disseminated histoplasmosis have chronic fever with molluscum-like skin lesions without accompanying infective endocarditis.

Our patient was given liposomal amphotericin *B* as soon as possible, as recommended by the Infectious Diseases Society of America in 2007. [16] Due to a mechanical obstruction of the mitral orifice, he underwent open-heart surgery, but due to refractory cardiogenic shock, he did not recover.

To date, there have been 5 reported cases of *H*. *capsulatum* endocarditis in Asia and Australia (Table 1). They were from Pakistan [17], Israel [20], Australia [18], and Thailand [19], (our patient). The male-to-female ratio was 4 : 1, with a mean age of 59 (range: 34–83) years. The comorbidity was noted in 2 (40%) patients. One [19] had the neurological sequence after septic cerebral emboli; another (our patient) had refractory heart failure. The duration of illness before diagnosis ranged from 1 to 4 months. The most common symptom was fever (5 patients, 100%), followed by dyspnea (2, 40%). Of the valve involved, there were 2 patients each with aortic and mitral valves and 1 patient with no reported valve. All except our patient had a prosthetic valve as a predisposition to infective endocarditis. Four of 5 patients had disseminated disease, while only one had isolated endocarditis. There were 2 categories of cutaneous manifestations including skin signs of endocarditis and those of disseminated infection. Two (40%) and 1 (20%) patients had skin signs of endocarditis and disseminated infection, respectively. The diagnosis was made by more than 1 method and clinical specimens. Blood cultures were positive for *H. capsulatum* in 1 (20%) patient. Fungal cultures from the vegetation in the premortem state were carried out in all patients. The treatment with both antifungal and heart surgery was performed in 4 patients. All except our patient recovered.

## 4. Conclusions

To date, there have been a handful of cases of *H. capsulatum* native valve endocarditis in non-HIV-infected patients. We report herein the first case in Thailand. Umbilicated skin lesions, especially combined with oral mucosal lesions, are a clinical clue that leads to the correct diagnosis of the causative organism.

## Figures and Tables

**Table 1:** A literature review of infective endocarditis caused by *Histoplasma capsulatum* in Asia and Australia.

Case number	Country/Year published	Sex/Age (year)	Clinical presentation		Echocardiogram	Diagnostic methodology	Treatment	Outcome
			Signs/Symptoms	Duration				
1.	Pakistan/2005 [17]	M/58	Low-grade fever	1 month	Vegetation at aortic prosthesis valve	Premortem	AMB 0.6 mg/kg/day plus AVR surgery and then itraconazole 400 mg/d	Recovered
			Disseminated (IE and bone marrow)			Serology (serum and urine)		
			No skin sign of IE			Culture (blood and valve)		

2	Australia/2011 [18]	M/83	Low-grade fever	1 month	Vegetation at aortic prosthesis valve	Premortem	AMB plus AVR surgery and then itraconazole	Recovered
			Disseminated (IE, liver, and spleen)			Serology (blood) and histology		
			No skin sign of IE			Culture (valve)		

3	Thailand/2013 [19]	F/58	Low-grade fever, dyspnea, and weight loss	2 months	2 large vegetations size, 2 × 0.9 cm and 1.1 × 0.7 cm, at mitral prosthesis valve	Premortem	AMB 1 mg/kg/day 6 weeks plus MVR surgery and then itraconazole 400 mg/d	Recovered, emboli to the brain
			No other organ involved			Histology		
			Splinter hemorrhage			Culture (valve)		

4	Israel/2013 [20]	M/64	Fever and night sweats	6 weeks	TEE showed no evidence of vegetations	Premortem	Itraconazole	Recovered
			Disseminated (lung, mediastinal node, and suspected IE)			Culture, PCR for fungus (mediastinal node)		
			Splinter hemorrhage					

5	Present study/2020	M/34	Low-grade fever and dyspnea	4 months	1.2 × 0.8 cm vegetation at mitral valve	Premortem	L-AMB 3.0 mg/kg plus AVR and MVR surgery	Death
			Disseminated (lung, skin, oropharynx, and IE)			Histology		
			No skin sign of IE			Culture (valve)		
